# Increase in excitability of hippocampal neurons during novelty-induced hyperlocomotion in dopamine-deficient mice

**DOI:** 10.1186/s13041-020-00664-8

**Published:** 2020-09-18

**Authors:** Masayo Fujita, Yukiko Ochiai, Taishi-Clark Takeda, Yoko Hagino, Kazuto Kobayashi, Kazutaka Ikeda

**Affiliations:** 1 Department of Psychiatry and Behavioral Sciences, Addictive Substance Project, Tokyo Metropolitan Institute of Medical Science, 2-1-6 Kamikitazawa, Setagaya-ku, Tokyo, 156-8506 Japan; 2 Neurology, Tokyo Metropolitan Neurological Hospital, 2-6-1 Musashidai, Fuchu-shi, Tokyo, 183-0042 Japan; 3 Department of Molecular Genetics, Institute of Biomedical Sciences, Fukushima Medical University, 1 Hikariga-oka, Fukushima-shi, Fukushima, 960-1295 Japan

## Abstract

Dopamine is involved in many important brain functions, including voluntary motor movement. Dysfunction of the dopaminergic system can induce motor impairments, including Parkinson’s disease. We previously found that dopamine-deficient (DD) mice became hyperactive in a novel environment 72 h after the last injection of L-3,4-dihydroxyphenylalanine (L-DOPA) when dopamine was almost completely depleted. In the present study, we investigated neuronal activity in hippocampal subregions during hyperactivity by measuring Fos expression levels using immunohistochemistry*.* Dopamine-deficient mice were maintained on daily intraperitoneal injections of 50 mg/kg L-DOPA. Seventy-two hours after the last L-DOPA injection, DD mice were exposed to a novel environment for 1, 2, or 4 h, and then brains were collected. In wildtype mice, the number of Fos-immunopositive neurons significantly increased in the hippocampal CA1 region after 1 h of exposure to the novel environment and then decreased. In DD mice, the number of Fos-immunopositive neurons gradually increased and then significantly increased after 4 h of exposure to the novel environment. The number of Fos-immunopositive neurons also significantly increased in the CA3 region and dentate gyrus in DD mice after 4 h of exposure to the novel environment. These results indicate that the delayed and prolonged excitation of hippocampal neurons in the CA1, CA3, and dentate gyrus that is caused by dopamine depletion might be involved in hyperactivity in DD mice.

Dopamine is involved in many important brain functions, including voluntary motor movement. Dysfunction of the dopaminergic system can induce motor impairments, including Parkinson’s disease. Therefore, locomotor activity is thought to be correlated with dopamine levels. We previously found that dopamine-deficient (DD) mice became hyperactive in a novel environment when brain dopamine levels were almost completely depleted [1], although DD mice were hypoactive in their home cage [2]. Dopamine deficiency in mice is induced by knockout of the tyrosine hydroxylase (TH) gene, with the concomitant restoration of TH expression under control of the dopamine β-hydroxylase (DBH) promoter to prevent the loss of epinephrine and norepinephrine [3]. Another research group also established a different DD mouse model and studied their behaviors. Their studies showed that DD mice exhibited deficiencies in motivated behavior, but they could still learn and express conditioned place preference for drugs ~ 24 h after L-DOPA administration [4]. However, these behaviors were not analyzed 72 h after the last L-DOPA injection. Hyperactivity in DD mice may be controlled in a dopamine-independent manner. We demonstrated the possibility of that a decrease in acetylcholine signaling might be involved in hyperactivity in DD mice in the previous study. However, the neuronal mechanisms of hyperactivity in DD mice are still unknown.

Rodents are hyperactive and express exploratory activity immediately after exposure to a novel environment, and then locomotor activity gradually decreases [5]. This decrease in locomotion occurs through intrasession habituation. In contrast, changes in locomotor activity in a novel environment is different in DD mice, which exhibit delayed exploratory activity and no intrasession habituation [1].

The hippocampus is important for locomotor control, the recognition of novelty, and spatial cognition [6–8]. Therefore, the present study investigated differences in neuronal activity in the hippocampus between wildtype (WT) and DD mice. We investigated neuronal Fos expression using immunohistochemistry in hippocampal subregions, including the CA1, CA3, and dentate gyrus (DG), which are known to be activated by exposure to novelty [9, 10].

DD mice were maintained on daily intraperitoneal injections of 50 mg/kg L-3,4-dihydroxyphenylalanine (L-DOPA; Nacalai Tesque, Kyoto, Japan). Seventy-two hours after the last L-DOPA injection, DD mice were exposed to a novel environment for 1, 2, or 4 h, and then brains were collected after perfusion with 4% paraformaldehyde (PFA; Nacalai Tesque). As a control, brain samples from WT mice were also collected. Brain samples from mice that were not exposed to a novel environment (0 h) were also collected. Brains were fixed overnight using 4% PFA, which was then replaced with 30% sucrose solution. Fixed brains were cut into 40 μm sections using a microtome with an attached electric specimen cooling device. The immunohistochemical analysis of free-floating sections was performed using rabbit polyclonal antibody (catalog no. 226003, Synaptic Systems, Goettingen, Germany). The signals were visualized with diaminobenzidine and observed under a light microscope. The number of Fos-immunopositive neurons in the CA1, CA3, and DG (− 2.0 mm from bregma) were counted using ImageJ software (National Institutes of Health, Bethesda, MD, USA). Statistical analyses were performed using two-way analysis of variance (ANOVA). Individual post hoc comparisons were performed using the Scheffe test. Values of *p* < 0.05 were considered statistically significant. The data were analyzed using BellCurve for Excel software (Social Survey Research Information, Tokyo, Japan).

In WT mice, a significant increase in Fos-expressing neurons was observed in the CA1 after 1 h of exposure to the novel environment, and then the number of Fos-expressing neurons gradually decreased (Fig. 1a). In DD mice, the number of Fos-expressing neurons was low after 1 h of exposure to the novel environment and then gradually increased after 2 and 4 h of exposure to the novel environment (Fig. 1a). The number of Fos-positive neurons in the CA3 and DG was also significantly higher in DD mice than in WT mice after 4 h of exposure to the novel environment (Fig. 1b, c). In WT mice, the number of Fos-immunopositive neurons was highest after 1 h of exposure to the novel environment and then decreased in the CA3 and DG, but this difference was not significant (Fig. 1b, c).

**Fig. 1:**
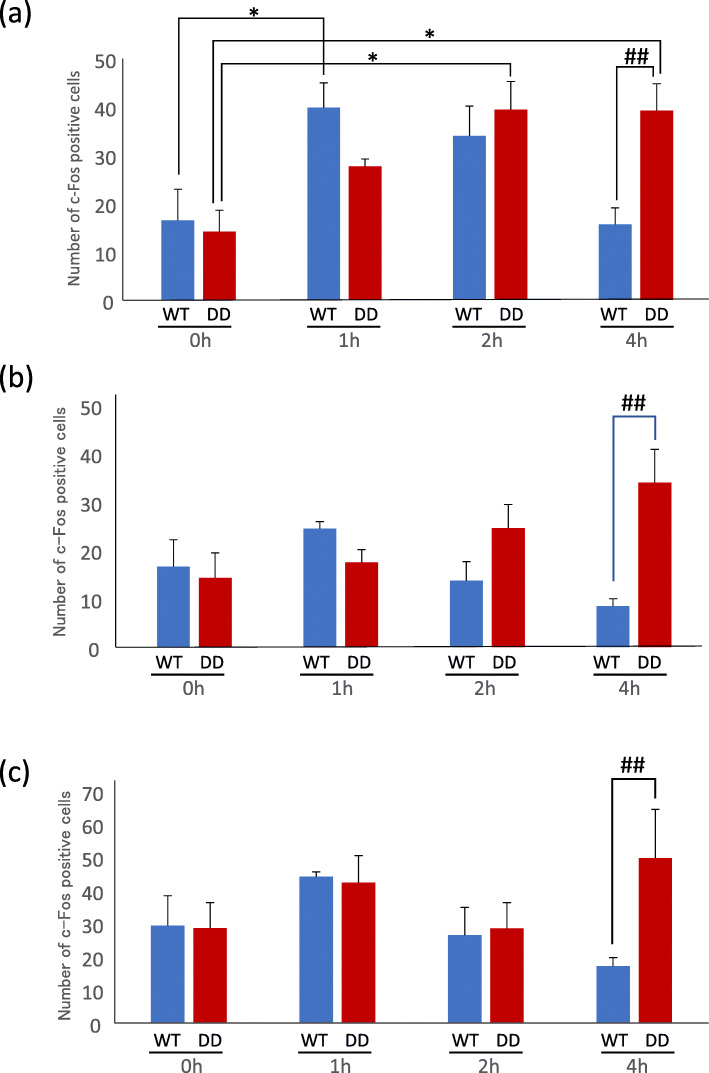
Number of Fos-positive neurons in the hippocampus before and after exposure to a novel environment. (**a-c**) Number of Fos-positive neurons (left) and representative images (right) from WT and DD mice after 0–4 h of exposure to a novel environment in the CA1 (**a**), CA3 (**b**), and DG (**c**) (*n* = 6/group). The data are expressed as mean ± SEM. **p < 0.01, compared with 0 h; ^##^*p* < 0.01, compared with WT mice. Scale bar = 200 μm

The increase in the number of Fos-positive neurons, especially in the CA1, was accompanied by an increase in locomotor activity in both WT and DD mice. Neuronal activity in the CA1 was previously reported to be involved in motor control [6]. The number of Fos-expressing neurons in the hippocampus was reported to increase when an animal was exposed to a novel environment [9], whereas the number of Fos-expressing neurons decreased after habituation [11]. Therefore, hippocampal activity may be involved in both locomotor control and novel environment recognition. In the present study, we focused on hippocampal neuronal activity because hyperactivity in DD mice was induced by novel environment exposure and because the hippocampus is one of the most important brain regions that process spatial novelty [9, 10]. Nonetheless, hyperactivity can also be controlled by other regions, such as the prefrontal cortex, striatum, and cerebellum. Neuronal activity in these additional brain regions should be studied in the future.

One issue is why Fos expression is delayed and prolonged in DD mice. Dopamine is upregulated during novelty recognition [12]. Additionally, treatment with a dopamine D_1_ receptor antagonist abolished the increase in Fos-expressing neurons after exposure to a novel environment [13]. These reports indicate that dopamine is important for novelty recognition. Dopamine is also important for spatial learning [12]. The hippocampus has two sources of dopamine: projections from dopaminergic neurons in the ventral tegmental area (VTA) and noradrenergic neurons in the locus coeruleus (LC) [14]. Tyrosine hydroxylase expression is rescued in noradrenergic neurons in DD mice. Even with a source of dopamine from the LC, total dopamine levels decrease in the hippocampus because of the lack of dopamine from the VTA in DD mice. The decrease in dopamine levels in the hippocampus might affect hippocampal activity and result in impairments in novelty recognition and spatial learning in DD mice.

In the previous study, we suggested that decreases in acetylcholine signaling may be involved in hyperactivity in DD mice. A muscarinic receptor agonist was reported to stimulate hippocampal activation [15]. Therefore, delayed hippocampal activation may be attributable to a decrease in acetylcholine signaling. The relationship between prolonged Fos expression and decreases in acetylcholine in DD mice may also be revealed using a muscarinic receptor agonist, such as oxotremorine M.

In conclusion, DD mice exhibited delayed and prolonged neuronal excitation in hippocampal subregions after exposure to a novel environment. These phenomena may be involved in hyperactivity in DD mice.

## Data Availability

The datasets used and/or analyzed during the current study are available from the corresponding author on reasonable request.
