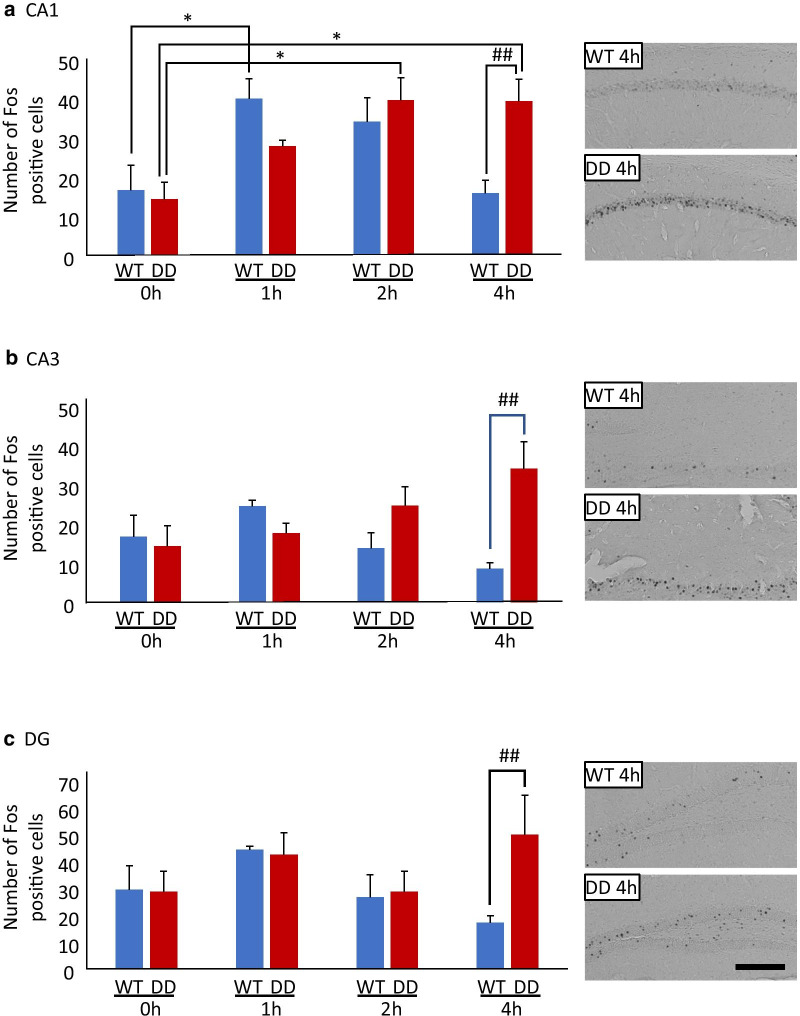# Correction to: Increase in excitability of hippocampal neurons during novelty-induced hyperlocomotion in dopamine-deficient mice

**DOI:** 10.1186/s13041-021-00741-6

**Published:** 2021-03-11

**Authors:** Masayo Fujita, Yukiko Ochiai, Taishi-Clark Takeda, Yoko Hagino, Kazuto Kobayashi, Kazutaka Ikeda

**Affiliations:** 1 Department of Psychiatry and Behavioral Sciences, Addictive Substance Project, Tokyo Metropolitan Institute of Medical Science, 2-1-6 Kamikitazawa, Setagaya-ku, Tokyo, 156-8506 Japan; 2 Neurology, Tokyo Metropolitan Neurological Hospital, 2-6-1 Musashidai, Fuchu-shi, Tokyo, 183-0042 Japan; 3 Department of Molecular Genetics, Institute of Biomedical Sciences, Fukushima Medical University, 1 Hikariga-oka, Fukushima-shi, Fukushima, 960-1295 Japan

## Correction to: Mol Brain (2020) 13:126 https://doi.org/10.1186/s13041-020-00664-8

Following publication of the original article [1], the authors identified an error in Fig. 1 and its caption. An incomplete version of Fig. 1 was published and a mistake was present in its caption. The incorrect and correct figure and its caption are published in this Correction article. The original article has been updated.

**Incorrect figure:**

**Fig. 1** Number of Fos-positive neurons in the hippocampus before and after exposure to a novel environment. **a**–**c** Number of Fos-positive neurons (left) and representative images (right) from WT and DD mice after 0–4 h of exposure to a novel environment in the CA1 (**a**), CA3 (**b**), and DG (**c**) (*n* = 6/group). The data are expressed as mean ± SEM. **p < 0.01, compared with 0 h; ^##^*p* 0.01, compared with WT mice. Scale bar = 200  μm


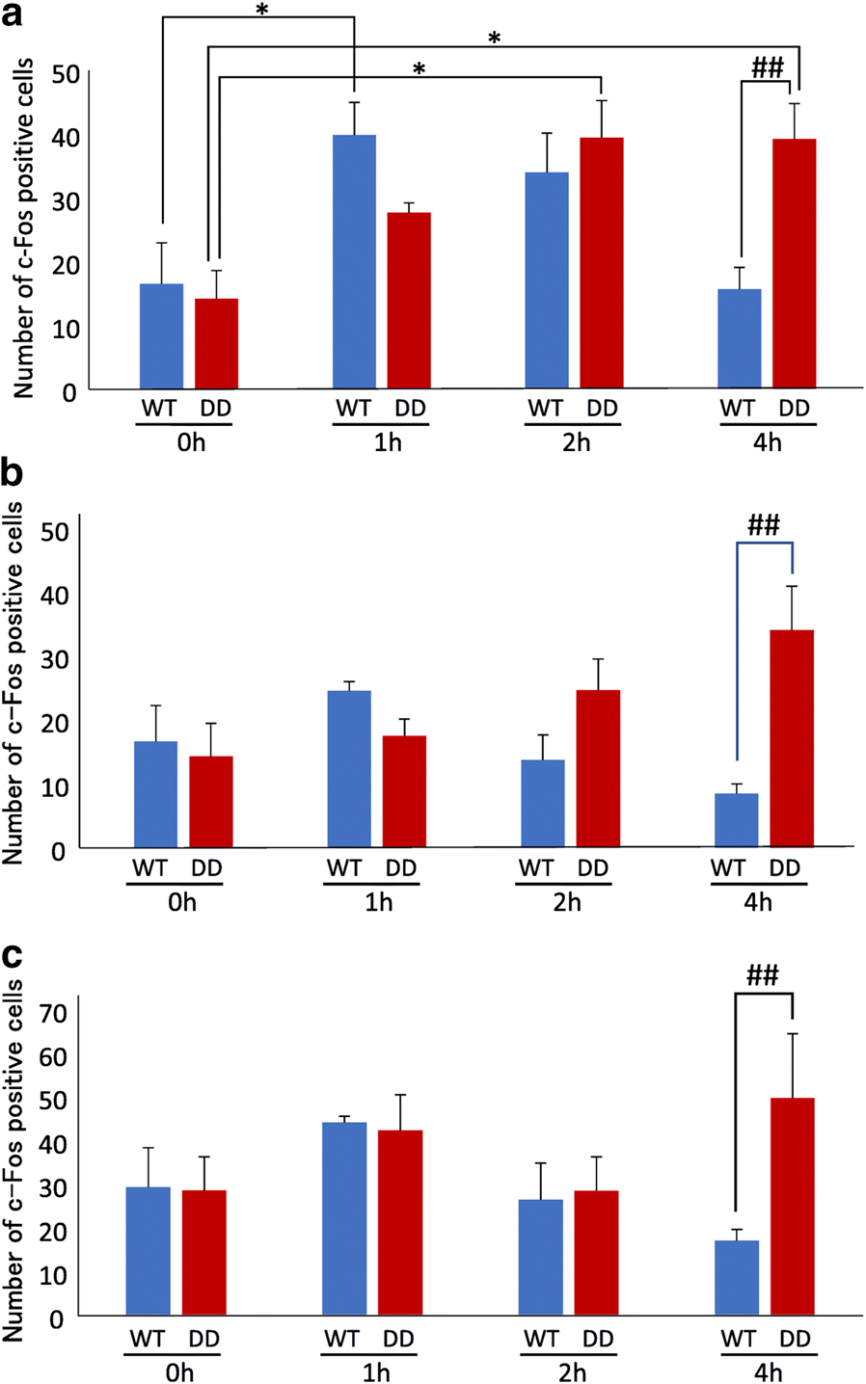


**Correct figure:**

**Fig. 1** Number of Fos-positive neurons in the hippocampus before and after exposure to a novel environment. **a**–**c** Number of Fos-positive neurons (left) and representative images (right) from WT and DD mice after 0–4 h of exposure to a novel environment in the CA1 (**a**), CA3 (**b**), and DG (**c**) (*n* = 6/group). The data are expressed as mean ± SEM. *p < 0.05, compared with 0 h; ^##^*p* < 0.01, compared with WT mice. Scale bar = 200 μm